# Complete Genome Analysis of the C4 Subgenotype Strains of Enterovirus 71: Predominant Recombination C4 Viruses Persistently Circulating in China for 14 Years

**DOI:** 10.1371/journal.pone.0056341

**Published:** 2013-02-18

**Authors:** Yan Zhang, Xiaojuan Tan, Aili Cui, Naiying Mao, Songtao Xu, Zhen Zhu, Jianhui Zhou, Jing Shi, Yueping Zhao, Xianjun Wang, Xueyong Huang, Shuangli Zhu, Yong Zhang, Wei Tang, Hua Ling, Wenbo Xu

**Affiliations:** 1 National Institute for Viral Disease Control and Prevention, Chinese Center for Disease Control and Prevention, Beijing, People’s Republic of China; 2 Jilin Provincial Center for Disease Control and Prevention, Changchun, People’s Republic of China; 3 Beijing Children Hospital, Capital University of Medical Science, Beijing, People’s Republic of China; 4 Anhui Provincial Center for Disease Control and Prevention, Hefei, People’s Republic of China; 5 Shandong Provincial Center for Disease Control and Prevention, Jinan, People’s Republic of China; 6 Henan Provincial Center for Disease Control and Prevention, Zhengzhou, People’s Republic of China; 7 Shanghai Center for Disease Control and Prevention, Shanghai, People’s Republic of China; 8 Chongqing Center for Disease Control and Prevention, Chongqing, People’s Republic of China; University of Hong Kong, Hong Kong

## Abstract

Genetic recombination is a well-known phenomenon for enteroviruses. To investigate the genetic characterization and the potential recombination of enterovirus 71 (EV71) circulating in China, we determined the 16 complete genome sequences of EV71 isolated from Hand Foot Mouth Disease (HFMD) patients during the large scale outbreak and non-outbreak years since 1998 in China. The full length genome sequences of 16 Chinese EV71 in present study were aligned with 186 genome sequences of EV71 available from GenBank, including 104 China mainland and 82 international sequences, covering the time period of 1970–2011. The oldest strains of each subgenotype of EV71 and prototype strains of HEV-A were included to do the phylogenetic and Simplot analysis. Phylogenetic analysis indicated that all Chinese strains were clustered into C4 subgenotype of EV71, except for HuB/CHN/2009 clustered into A and Xiamen/CHN/2009 clustered into B5 subgenotype. Most of C4 EV71 were clustered into 2 predominant evolutionary branches: C4b and C4a evolutionary brunches. Our comprehensive recombination analysis showed the evidence of genome recombination of subgenotype C4 (including C4a and C4b) sequences between structural genes from genotype C EV71 and non-structural genes from the prototype strains of CAV16, 14 and 4, but the evidence of intratypic recombination between C4 strains and B subgenotype was not enough strong. This intertypic recombination C4 viruses were first seen in 1998 and became the predominant endemic viruses circulating in China mainland for at least 14 years. A shift between C4a and C4b evolutionary brunches of C4 recombination viruses were observed, and C4a viruses have been associated with large scale nationwide HFMD outbreak with higher morbidity and mortality since 2007.

## Introduction

The Enterovirus genus in the family Picornaviridae consists of 4 species with strains isolated from humans: Human enterovirus A (HEV-A), HEV-B, HEV-C, HEV-D [1]. Human enterovirus 71 (EV71) is one of the member in HEV-A species. The genome of enteroviruses consists of a single-stranded positive-sense RNA of approximately 7400 nucleotides. The viral genome contains a 5′- and 3′-untranslated regions (UTRs) which are essential for viral RNA replication. The genome is translated as a single large polyprotein that is composed of four capsid proteins, VP1 to VP4, and seven nonstructural proteins, 2A, 2B, 2C, 3A, 3B, 3C, and 3D [2]. VP1 to VP4 capsid proteins were encoded by P1 region. The P2 (2A, 2B, 2C) and P3 (3A, 3B, 3C, and 3D) regions encode nonstructural proteins involved in polyprotein processing, RNA replication and shut-down of host-cell protein synthesis. The RNA-dependent RNA polymerase, 3D, is a major component of the viral replication complex which also include other viral proteins, such as 2BC, 2C, 3AB, and 3Cpro [3].

EV71 infection, which was first reported in the USA, has been a recurrent feature in the Asia-Pacific region since its first outbreak in Sarawak, Malaysia in 1997 [4]. In recent years, numerous large outbreaks of EV71-associated Hand Foot Mouth Disease (HFMD) with high morbidity and mortality have occurred in Asian countries and regions, including Singapore [5], South Korea [6], Malaysia [7], Japan [8], Vietnam [9], Mainland China [10], [11], and Taiwan [12]. This phenomenon has increased the research interest in EV71, leading to extensive nucleotide sequencing and genotype description [13], [14]. Based on VP1 coding region, EV71 is divided into four genotypes: A, B, C and D [15], and within the genotypes B and C, there are further subgenotypes, B0–B5 and C1–C5 [10], [14], [16].

In China, large scale EV71 outbreak of HFMD associated with acute neurological disease occurred in 2007 at Linyi City, Shandong province [10], and since then the outbreak pattern has repeated and exacerbated year by year, with increasing morbidity and mortality [11], [17]. It has been confirmed that subgenotype C4 has been the sole viral genetic lineage circulating in mainland China since 1998 [17]. The large HFMD outbreaks with fatal neurological complications that have occurred since 2007 are mainly due to subgenotype C4a of EV71 [10], [11], [17].

Genomic recombinations are well known to contribute to genetic variations and evolution of enteroviruses. Complete genome analysis of prototype HEV-A indicated that recombination in the nonstructural region has played a role in the evolution of some HEV-A prototypes [18]. Phylogenetic analyses of several available sequences of EV71 have shown that recombination occurred between EV71 and coxsackievirus A16 in the nonstructural region [19], [20] and between different subgenotypes of EV71 viruses [21].

Our previous studies have been confirmed that the large-scale HFMD outbreaks with fatal neurological complications that have occurred since 2008 are mainly due to subgenotype C4a of HEV71, which was identified as a recombination virus with CVA16 in 3D region [11]. And as these recombination viruses associated outbreaks might be a threat to public health in China, it would be worthwhile to perform an extensive genetic analysis for the full length genome of C4 viruses circulating in mainland China during a period covering both before and after the large scale outbreaks in order to understand the increase of the scale of the outbreak and the severe cases in mainland China in recent years.

The intensive surveillance for HEV71 circulation maintained by mainland China during and after the 2007 outbreak permitted a detailed analysis of a large number of isolates from the HFMD patients by complete genome analysis methods. To investigate the genetic characterization of complete genome of C4 subgenotype EV71 strains and the recombination with prototype strains of HEV-A strains, we performed a large scale genomic sequence analysis of isolates (n = 202) collected from 17 countries worldwide over a 4 decades period. We sequenced and analyzed the entire genome sequences of 16 C4 subgenotype EV71 isolated from HFMD or encephalomyelitis or fatal patients during a period of both before and after EV71 large-scale outbreak in mainland China. The phylogenetic analysis, similarity plot and bootscan analysis were performed to analyze the phylogenetic relationship and potential recombination between C4 subgenotype EV71 strain circulating in mainland China, oldest strain of EV71 subgenotype and other prototype stains of HEV-A species.

## Results

### Genetic Characterization of Full Length Genome of Chinese Mainland EV71

**Figure 1:**
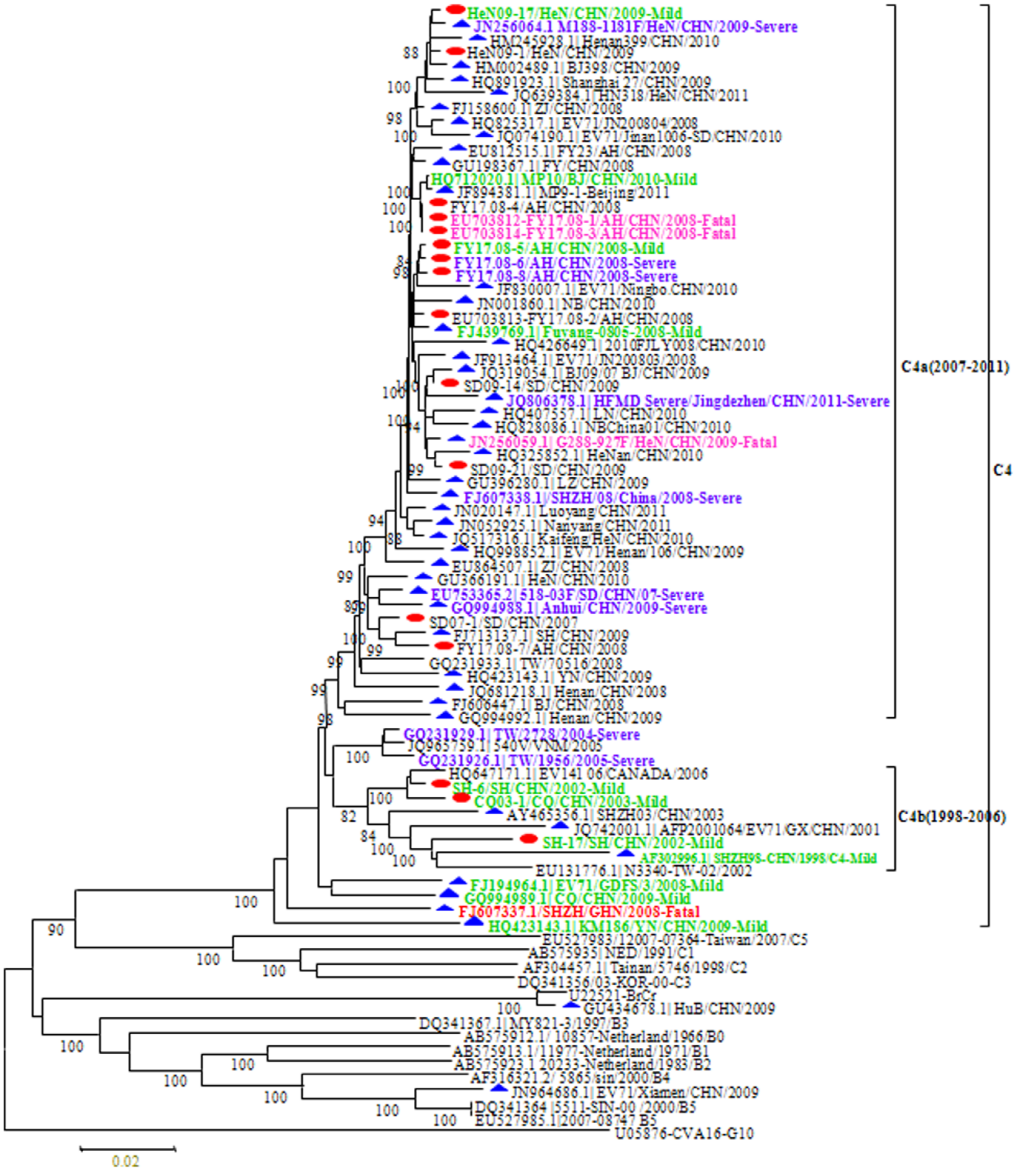
Phylogenetic tree based on complete genome of Chinese mainland EV71. Red dots indicate the sequences from Mainland China and blue triangles indicate the sequences downloaded from GenBank. The prototype of EV71(BrCr), CVA-16(G-10) and the oldest sequences of different subgenotypes of EV71(B0-5, C1-5 subgenotypes) were included as well. Green color strain name indicate the mild case, purple color strain name indicate the severe case and pink color strain name indicate the fatal case.

The full length genome sequences of 16 China EV71 in present study were aligned with 186 genome sequences of EV71 available from GenBank, including 104 China mainland and 82 international sequences from South and East Asia(22), Australia(8), and Europe(8), America (10), Japan(9), Korea(2), Taiwan(23), and the collection date of the clinical specimens of 186 sequences covered from 1970–2011(Table S1). Similar to other reported genomes of EV71, the full length of 16 China EV71 was 7404–7406 nucleotides. The difference of genome length resulted from insertion or deletion in the 5′ untranslated region (UTR) of 741–743 nucleotides. No deletions and insertions were observed in the P2, P3 and 3′ UTR genomic regions, which were composed of a single open reading frame(ORF) of 6,579 nucleotides encoding a polyprotein of 2,193 amino acid and a 3′ UTR of 82 nucleotides preceding the poly(A) tract. Nucleotide substitutions among different EV71 strains were scattered throughout the genome. The sequence homology of nucleotide acid and deduced amino acid between 16 China EV71 viruses was 93.5%–100% and 83.7%–100%, respectively. It is noteworthy that, compared with all C4a HEV71, a nucleotide substitution in all C4b HEV71 genome (A to C reversion at nt2503 in the VP1 coding region, which caused amino acid substitution of VP1–10: Gln to His) had reverted. Phylogenetic analysis of all the Chinese EV71 based on the complete genome showed that all strains were clustered into C4 subgenotype group, except for HuB/CHN/2009 clustered into A and Xiamen/CHN/2009 clustered into B5 (Fig. 1). Xiamen/CHN/2009 is the first B5 isolate found in China mainland. Most of C4 EV71 were clustered into 2 predominant evolutionary branches: C4b and C4a.C4a lineage was composed of the EV71 strains circulating in mainland China during 2007–2011, and this indicated that C4a lineage Chinese strains evolved independently from other C4 viruses. C4b lineage included Chinese older viruses back to 1998 and fewer international strains before 2007. Taiwan strains circulating in 2004–2005 were grouped into a minor brunch with Vietnam strain independent from mainland Chinese strains. It indicted that Taiwan and Vietnam strains evolved independently from those viruses circulating in mainland of China. 4 orphan (single sporadic virus forms single brunch in the phylogenetic tree) strains from mainland of China have quite different diversity with other C4 viruses, suggesting these single sporadic isolates probably imported rather than derived from the main predominant evolutionary branches of other mainland Chinese strains (Fig. 1).” 16 Chinese EV71 shared 93.5–100% nucleotide sequence identities with each other. Phylogenetic analysis based on the different region of the C4 EV71 strains showed all C4 EV71 strains were clustered into the same group separated from other strains of human enterovirus A species in the region of 5′UTR, P1, P2 and P3. However, the C4 EV71 was clustered with CVA-16, CVA-14 and CVA-4 closely in the region of 3′UTR (tree not shown), might because of the short sequence window. HeN09-17/HeN/CHN/2009-C4a and SH-17/SH/CHN/2002-C4b were chosen as the representative strain for C4a and C4b, respectively. These two representative strains were used to do the further phylogenetic and recombination analysis.

### Nucleotide Acid and Deduced Amino Acid Sequence Identities Analysis Between C4a, C4b and the Oldest Strain of Each Subgenotype of EV71 and Other Prototype Strains of HEV-A Species

A comprehensive comparison of nucleotide acid and deduced amino acid sequence identities between C4a, C4b and the oldest strain of each subgenotype of EV71 and other HEV-A species prototype strains is shown in Table 1. C4a and C4b viruses shared the 74.9–89.5% homology with BrCr strain, representative strains of other EV71 subgenotype and other HEV-A species in the region of 5′UTR. We found that both C4a, C4b viruses had much higher sequence identities with BrCr and other EV71 (nucleotide acid: 80.4–91.2%; amino acid: 62.4–100%) than other HEV-A species strains(nucleotide acid: 56.9–72.3%;amino acid:37.9–78.7%) in the region of overall capsid protein P1 sequences and sequences of the individual mature proteins, VP4 to VP1. Interestingly, in the P2 and P3 regions, the nucleotide acid sequence identities between C4a, C4b viruses and BrCr (nucleotide acid: 77.1–78.2%; amino acid: 95.4–96.1% ) was lower than that between C4a, C4b viruses and CVA-16,14,4 (nucleotide acid: 81.8–84.6%; amino acid: 96.5–97.7%), especially in the region of 2A, 2B, 2C, 3B, 3C, 3D. The 3′UTR sequences of the C4a, C4b viruses analyzed were similar to those of the other EV71 and more than 61.8% identical to each other but the identities varied distinctly from the prototype strains of different other HEV-A viruses (varied from 29.4% to 70.6%) (Table 2).

**Table 1:** List of the HEV-A strains used to generate the identities, phylogenetic and recombination analysis.

Abbreviation	Strain name	Genbank Accession Number	The place of isolation	The year of isolation	Genotype/Subgenotype	prototype or representative strains
EV71	10857/NED/1966	AB575912	Netherlands	1966	B0	oldest
EV71	11977/NED/1971	AB575913	Netherlands	1971	B1	oldest
EV71	20233/NED/1983	AB575923	Netherlands	1983	B2	oldest
EV71	MY821-3/1997	DQ341367	Singapore	1997	B3	oldest
EV71	5865/sin/000009/SIN/2000	AF316321	Singapore	2000	B4	oldest
EV71	5511-SIN-00	DQ341364	Singapore	2000	B5	oldest
EV71	NED/1991	AB575935	Netherlands	1991	C1	oldest
EV71	Tainan/5746/98/TW/1998	AF304457	Taiwan	1998	C2	oldest
EV71	06-KOR-00/KOR/2000	DQ341355	South Korea	2000	C3	oldest
EV71	SHZH98/CHN/1998	AF302996	China	1998	C4	oldest
EV71	2007-07364/TW/2007	EU527983	Taiwan	2007	C5	oldest
EV71	BrCr/USA/1970	U22521	USA	1970	A	prototype
COXA2	Fleetwood/USA/1947	AY421760	USA	1947	A2	prototype
COXA3	Olson/USA/1948	AY421761	USA	1948	A3	prototype
COXA4	High Point/USA/1948	AY421762	USA	1948	A4	prototype
COXA5	Swartz/USA/1950	AY421763	USA	1950	A5	prototype
COXA6	Gdula/USA/1949	AY421764	USA	1949	A6	prototype
COXA7	Parker/USA/1949	AY421765	USA	1949	A7	prototype
COXA8	Donovan/USA/1949	AY421766	USA	1949	A8	prototype
COXA10	Kowalik/USA/1950	AY421767	USA	1950	A10	prototype
COXA12	Texas-12/USA/1948	AY421768	USA	1948	A12	prototype
COXA14	G-14/SOA/1950	AY421769	Republic of South Africa	1950	A14	prototype
COXA16	G10/SOA/1951	U05876	Republic of South Africa	1951	A16	prototype

**Table 2:** Pairwise amino acid sequences identities between Chinese representative strains of EV71 and other prototype strains of HEV-A species.

Region	%identity											
	C4a						C4b					
	BrCr	Other EV71*	CA16	CA14	CA4	Other HEV-A	BrCr	Other EV71*	CA16	CA14	CA4	Other HEV-A
5′UTR^a^	86.7	85.7–88.7	85.0	87.0	86.8	74.9–88.3	87.1	87.0–89.5	85.7	87.3	87.5	74.9–88.6
P1	73.2	73.7–86.9	48.8	40.6	41.1	35.3–38.2	73.0	73.3–87.9	47.8	40.6	39.0	33.9–37.2
VP4	100.0	85.5–100.0	75.5	52.7	61.6	57.3–63.7	100.0	85.5–100.0	75.5	52.7	61.6	57.3–63.7
VP2	64.5	66.0–78.4	41.5	45.9	45.9	31.2–49.2	62.4	59.5–83.8	37.9	47.6	45.0	30.2–47.6
VP3	89.2	88.1–93.2	77.6	66.4	66.4	59.7–69.0	90.2	89.7–94.2	78.7	67.1	65.1	58.3–69.0
VP1	73.2	73.7–92.0	48.8	40.6	41.1	35.3–41.6	73.0	73.3–87.9	47.8	40.6	39.0	33.9–39.6
P2	95.4	95.2–98.1	97.4	97.0	96.5	76.2–96.8	96.1	95.6–98.4	97.7	97.7	97.5	76.7–97.4
2A	94.5	93.8–98.0	95.9	95.2	94.5	62.4–95.2	95.9	94.5–98.0	95.9	96.6	95.9	63.4–96.6
2B	93.7	90.5–98.0	98.0	98.0	96.9	70.9–96.9	94.8	91.6–99.0	99.0	99.0	98.0	77.2–98.0
2C	96.3	94.7–98.2	97.8	97.5	97.2	82.7–97.8	96.6	99.1–95.0	98.2	97.8	98.2	82.4–98.2
P3	92.5	92.7–97.1	97.5	97.3	97.1	79.5–94.8	92.0	92.5–97.5	97.4	97.7	97.1	79.0–94.5
3A	97.6	91.3–98.8	97.6	98.8	98.8	64.7–98.8	96.4	90.0–97.6	96.4	97.6	97.6	63.0–97.6
3B	31.6	31.6–73.7	47.4	57.9	63.2	21.1–47.4	31.6	36.8–78.9	63.2	78.9	84.2	31.6–57.9
3C	92.5	91.9–97.7	97.2	97.7	97.7	80.3–96.0	92.5	93.1–98.9	98.3	98.9	98.9	80.3–96.0
3D	91.8	92.7–96.9	98.0	97.1	96.9	81.5–94.4	91.3	92.3–97.3	97.6	97.6	96.7	81.5–93.9
3′UTR^a^	67.6	64.7–76.5	70.6	76.5	79.4	29.4–70.6	67.6	61.8–64.7	70.6	70.6	73.5	29.4–70.6

* not including the prototype strain of C4 subgenotype: SHZH98 a nucleotide acid sequence identities between Chinese representative strains of EV71 and other prototype strains of HEV-A species.

### Phylogenetic Analysis of the Chinese C4 EV71 Strains and Other HEV-A Genomes

**Figure 2:**
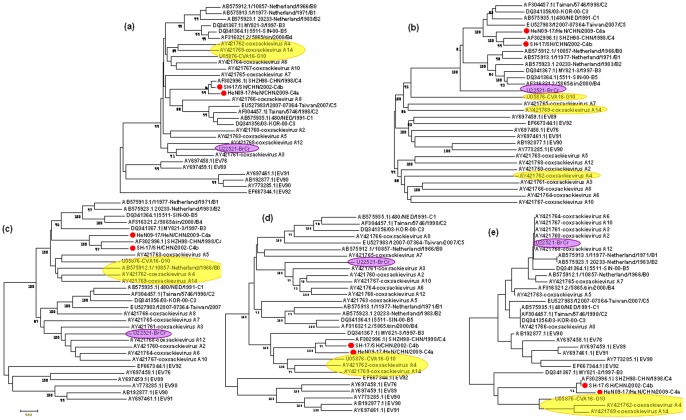
Rooted phylogenetic trees showing the relationships amongst HEV-A isolates using the different genomic regions. The neighbour-joining trees were constructed from alignment of the 5' UTR (a), P1 (b), P2 (c), P3 (d) and 3′UTR (e) genomic region, respectively. The percentage of bootstrap (percentage of 1000 pseudoreplicate datasets) replicates supporting the trees are indicated at the nodes; for clarity, only values over 80% are shown. The branch lengths are proportional to the genetic distances corrected using Kimura-two-parameter substitution model.

To investigate the genetic relationship between the Chinese C4 EV71 strains and the oldest strains of EV71 subgenotypes and other prototype HEV-A strains available in GenBank, the phylogenetic trees based on the full length of genome and 5′UTR, P1, P2, P3, 3′UTR region of the genome were constructed respectively (Fig. 2a–e). At the full length of genome and P1 region, there was similar pattern of the phylogeny between the Chinese C4 EV71 strains and oldest strains of EV71 subgenotypes and other prototype HEV-A strains: the Chinese C4 EV71 strains were clustered with C subgenotype of EV71 closely, and segregated away from other prototype HEV-A strains. At the region of 5′UTR, all members of HEV-A are closely related to one another (Fig. 2a). At both P2 and P3 genomic region, the Chinese C4 EV71 strains were phylogenetically closer to CVA-16, CVA-14, CVA-4 prototype strains than to the EV71 prototype strain BrCr (Fig. 2c,2d). At 3'UTR, C4 EV71 isolates were clustered with EV71 subgenotype of B3 and CV-A4, CV-A14 and CV-A16 (95% bootstrap support).

If we performed the phylogenetic analysis based on the detailed coding region of P2 and P3, we found a slightly different phylogenetic relationship between C4 subgenotype and other subgenotype of EV71 and other HEV-A prototype strains (Fig. S1). C4a, C4b and the prototype strain of C4 subgenotype consistently clustered together with each other in all the region of P2 and P3, except for at the region of 2B, where C4a, C4b segregated from the prototype strain of C4 subgenotype, shzh98/CHN/1998, and cluster with B genotype and some HEV-A strains. At the region of 2A, Chinese C4 viruses were phylogenetically closest to C genotype strains and CVA-4 prototype strain; at 2B region, C4a, C4b viruses closest to CVA-4; This indicated that 2A, 2B region was a genomic region of multi-mutation and a breakpoint for the recombination; while at 2C,3A,3B region, similar phylogeny pattern was shared between C4a, C4b viruses and other HEV-A strains: C4a, C4b viruses were closest to B0-5 subgenotype strains, and trended to closer to CVA-16, CVA-14, CVA-4; At 3C, 3D and 3′UTR region, the C4a, C4b viruses apparently to be closer to CVA-16, CVA-14,CVA-4 than the subgenotypes strains of B genotype, except for B3 subgenotype. Isolates of subgenotype C4 were clustered with B3 isolates, CV-A4, CV-A14 and CV-A16 and support for the clustering was also significant (100% bootstrap). Therefore, the phylogenetic relationships of the viruses were different with respect to different positions in the genome. The observed differences in the phylogenetic tree topologies between the capsid and the noncapsid regions indicate that recombination might have occurred during the evolution of these viruses.

### Recombination Analysis of the Chinese C4 EV71 Strains and Other HEV-A Genomes

**Figure 3:**
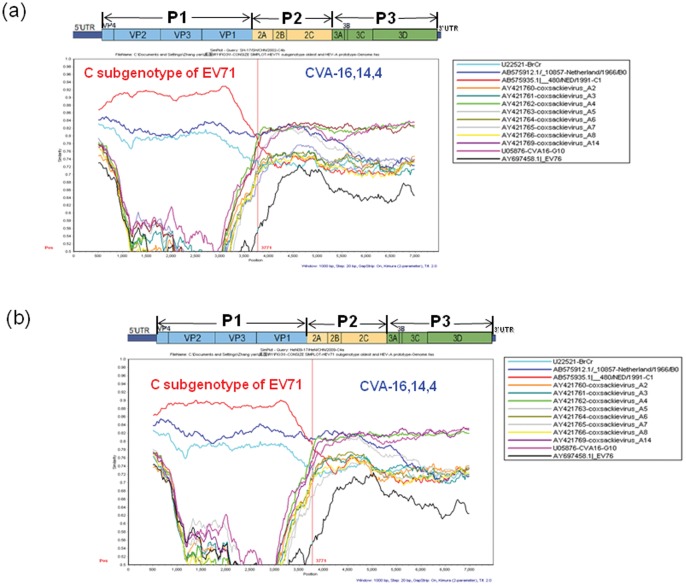
Simplot analyses of the C4a, C4b and the oldest strains of subgenotypes of EV71 and other HEV-A prototype strains on the basis of full-length genomes. Each analysis used each of the two lineages viruses as the query sequence. A sliding window of 1000 nucleotides moving in 20-nucleotide steps was used in this analysis. (a) C4b virus: SH-17/SH/CHN/2002; (b) C4a virus: HeN09-17/HeN/CHN/2009.

The genome sequences of Chinese C4 subgenotype viruses and all available HEV-A prototype strains were analyzed with Simplot software, using the representative strain of each lineage in turn as the query sequence. Similarity plot analyses demonstrated that C4a, C4b viruses showed the highest degree of similarity to the C genotype of EV71 in the capsid region, but in the non-capsid region, C4a, C4b viruses were all contained an unidentified sequence in the P2 and P3 coding region that was apparently not related to those of EV71 strains (Fig. 3). Comparison of the P2 and P3 coding region sequences of the C4a, C4b EV71 strains with those of the certain prototype strains of HEV-A, B, C, and D revealed no sequence match above 85.8%, and showed higher similarity to HEV-A than to HEV-B, C, and D. In addition, the deduced amino acid sequence of the recombinant noncapsid sequences of the C4a and C4b EV71 strains showed a high identity with HEV-A, especially those of prototype CVA16 (>97.4%), prototype CVA14 (>97.0%), and prototype CVA4 (>96.5%).

Subsequent bootscan analyses indicated possible recombination events. C4a, C4b EV71 strains were most closely related to the C genotype of EV71 in the 5′ half of the genome, which is consistent with the Simplot analysis results. However, after the junction sequences between VP1 and 2A, the bootscan graph exhibited a sound phylogenetic relationship between C4a, C4b EV71 strains and CVA-16, 14, 4.

Support for the inter-typic recombination involving 3 prototype strains of HEV-A and EV-71 were demonstrated by one major breakpoint identified in nucleotide 3700–3826 within the 2A gene of the complete genome (Fig. 3), where switching the C4a and C4b genome sequences to that of the non-structural of CVA-16, 14, 4. Although this nucleotide 3700–3826 breakpoint also occurred between C genotype and B genotype of EV71, the B genotype strains keep consistent similarity with the query strain of C4a and C4b viruses. This analysis indicated that there is no strong evidence to support the conclusion of the intratypic recombination between C and B subgenotypes of EV71.

4 orphan strains of C4 subgenotype, FJ194964-EV71/GDFS/3/2008, GQ994989-CQ/CHN/2009, FJ607337-SHZH/CHN/2008Fatal, HQ423143-km186/yYN/CHN/2009, were performed with Simplot analysis. A strain from the fatal case, FJ607337-SHZH/CHN/2008Fatal, shared the similar homology (>94%) with other 3 orphan strains from non-fatal cases in 5′UTR, P1 and P2 region of the genome, but the homology decreased gradually in the P3 region down to 85% in 3D region(not shown). It has been reported that sequences in the viral RNA-dependent RNA polymerase 3D (3Dpol) gene are important in determining the neurovirulence of polioviruses [22], [23]. Similarly, mutation of the EV71 standard strain BrCr in the 3Dpol region, which catalyzes both (−)-strand and (+)-strand RNA syntheses, showed attenuated neurovirulence in the cynomolgus monkey model [24]. Whether this variation in the 3D region *play an important role* for the death of the fatal case needs to be determined by further study.

### Transmission and Evolution of C4 Recombination Virus in China Since 1998

C4 recombination virus was first isolated in ShenZhen, Guangdong province of China in 1998 [25]. Our present study indicated that the C4 recombination viruses have been the predominant endemic viruses circulating in China mainland for at least 14 years since 1998. During the epidemic of the C4 recombination viruses, it evolved into 2 major evolution brunches, C4a and C4b viruses in the decade. Our present and previous data indicated that C4b viruses had been circulating in mainland China for 5 years since 1998 (C4b was prevalent from 1998 to 2003, but its transmission was interrupted since 2003), which rarely caused severe disease and death in HFMD patients, and replaced by C4a viruses causing the nationwide outbreak with higher morbidity and mortality since 2007, indicating a shift between 2 evolutionary brunches within subgenotype C4 recombination viruses. A total of 5,034,764 (range from: 489,082-1,619,148 each year) HFMD cases including 61,582(range from 1,164-27,891 each year) severe and 1,894(range from 126-904 each year) fatal cases were reported to NNDRS during 2008-2011 in mainland of China [11], [17], with increase of the severe and fatal cases year by year. The recombination C4a EV71 viruses have been associated with more than 80% of the severe cases and 92% of the fatalities since 2008 [17].

For the geographical transmission of the recombination C4 viruses, we found that C4b viruses circulated in southern provinces of China, such as, Guangdong, Chongqing, Shanghai,Guangxi; and C4a viruses transmitted extensively throughout.

## Discussion

The phylogenetic analysis of complete genome of EV71 circulating in mainland China in this present study showed all Chinese strains were clustered into C4 subgenotype group, except for HuB/CHN/2009 clustered into A and Xiamen/CHN/2009 clustered into B5(Fig. 1). Our previous studies on EV71 epidemiology showed that reported EV71 infections in mainland China had been associated with the only predominant subgenotype C4 viruses for more than 10 years, except for 2 orphan C genotype viruses were found in 1987 and 1997 respectively. The C4 subgenotype HEV71 has been the predominant endemic viruses circulating in mainland of China since 1998. However, there has been a shift of multiple subgenotypes of HEV71 circulating in other area, such as Taiwan, Australia and Malaysia. The patterns of HEV71 prevalence varied among different areas. Maybe because of the large and high density population in mainland of China, there is large newborn cohort every year becoming the susceptible population; therefore, the endemic viruses could be circulating in mainland China persistently for many years. While in other areas with small and low density population, the endemic viruses could be interrupted after circulating for a period of time with the increase of the population immunity, and then, other genotypes/subgenotypes HEV71 could be imported and becoming the endemic strains. It is also interesting that the molecular epidemiological pattern of HEV71 in mainland China appears to be similar to those of the measles virus and CVA-16 [26], [27], a single predominant endemic virus circulated in mainland China for a long time; but to be quite different from those of the rubella virus that different genotypes have co-circulated in China [28].

In this study, one B5 subgenotype virus was found in Xiamen (located in south of China) in 2009, which is the first B5 isolate found in China mainland. B5 subgenotype viruses were first found in Japan and Malaysia in 2003 [29] and later on circulating in other country or region, such as, Vienam [9] and Taiwan [12].Genotype A is composed of the EV71 strain (BrCr-CA-70) identified in 1970 in the USA but was not detected afterwards until 2008 [13], [30]. Yu et al [30] reported the emergence of five isolates that are closely related to genotype A in central China. This was the first report of the occurrence of the modern contemporary A genotype EV71. The sequence homologies between HuB/CHN/2009 A strain and the prototype BrCr was 98.9%-98.0% for nucleotide acid and amino acid, respectively, in the full length genome. Based on the reported evolution rates calculated for HEV71 [13], 3.18×10^−3^ nucleotide substitutions/site/year, the diversity between the oldest BrCr found in 1970’s and modern Chinese strains found in 2008–2009 would be at least 18%. Therefore, the occurrence of both HuB/CHN/2009 and Luan/CHN/2008 appeared weird. These A genotype viruses might be the laboratory adapted strains or the laboratory contamination. Although C4 subgenotype was the predominant strains circulating in mainland China for more than 10 years, the occurrence of orphan viruses of other genotype or subgenotypes, C, B5 and weird A, indicate the requirement of extensive surveillance of EV71 in mainland China should be strengthened.

No reported severe or fatal cases caused by C4b viruses occurred before large scale outbreak of HFMD in mainland China during 1993–2003 based on our analysis of complete genome. However, during the large scale outbreak of HFMD, increasing neurovirulence associated with C4a virus is a big concern for public health in mainland China. Our study on the analysis of complete genome, C4a viruses caused different phenotype of disease from mild to fatal. No specific lineages were associated with severe or fatal or mild cases. This indicated the viruses isolated from different phenotypic patients derived from the common ancestor and evolutes to different lineages by mutating gradually. We speculated that both viruses and host factor contributed to the phenotype of the disease.

Genomic recombinations are well known to contribute to genetic variations and evolution of enteroviruses. A range of enteroviruses of various serotypes or genotypes co-circulating in populations at some point of time was reported by different research groups [31]. In the period of the enterovirus co-circulation, recombination between parts of genome of different serotype or genotype viruses may occur when different viruses infect and replicate in the same cell. This recombination process allows enteroviruses to create and maintain their genetic diversity and fitness. Several reports have shown their evidences for EV71 recombination, including intertypic and intratypic recombination between subgenotypes of EV71 and other prototype strains of HEV-A [20], [21], [32]. But these studies reported the intratypic recombination between genotype/subgenotypes of EV71 based on the analysis of consensus sequences of genotype/subgenotypes viruses or the representative strains of genotype/subgenotype, not based on the oldest strain within the genotype or subgenotype group, which is the very important for the recombination analysis. In this study, we download all the complete genome sequences of EV71 from GenBank and searched by the published paper or the sequence submission information for the oldest strain within each subgenotype group of EV71, and then, the C4a and C4b viruses were aligned with the oldest strains of each subgenotype of EV71 and the prototype strains of HEV-A. Our comprehensive recombination analysis showed the evidence of genome recombination of genotype C4 (including C4a and C4b) sequences between EV71 genotype C structural genes and non-structural genes derived from the prototype strains of CAV16, 14 and 4, but the evidence of intratypic recombination between C4 strains and B subgenotype was not enough strong, since the B subgenotype showed the consistent high homology (80–86%) with C4a and C4b strains in 5′ end genome sequence(5′UTR-P2), while in the P3 region, higher homology was found between C4a, C4b strains and the prototype strains of CAV16, 14, 4, not B subgenotype EV71. In summary, these analyses showed the evidence of genomic recombination of C4a and C4b sequences between EV71 genotype C structural genes and non-structural genes derived from CAV16, 14, 4, not from B subgenotype EV71. This finding was inconsistent with the previous study [20], [21], [32](Table S3).

Interestingly, the EV-71 isolates of subgenotype B3 shared the similar recombination pattern with C4. These isolates had high sequence similarity to EV-71 genotype B, CV-A4, CV-A14 and CV-A16/G10 at P2 genomic region (≥81%) and high sequence similarity to CV-A4, CV-A14 and CV-A16 at P3 genomic region ((≥83%), Figure 2). At the P3 genomic region, the sequence similarity of isolates of subgenotype B3 and C4 to the rest of the EV-71 genotypes was only between 75–79%. In the phylogenetic tree, the B3 strains consistently clustered with B1,2,4,5 in the genome sequences except for P3 region, where B3 segregated from other B subgenotype and clustered with CV-A4, CV-A14 and CV-A16 more closely. It is indicating that B3 shared the similar evolution pattern with C4 subgenotype viruses from the common ancestor: they likely ‘‘trap’’ sequences from other HEV-A viruses, thereby producing new individual viruses that differ from the parental strains during natural multiplication of HEV71 strains.

The clustering of isolates of subgenotype C4 with B3 and CVA4, 14, 16 at the 3' UTR genomic region was consistent with the previous clustering at the P2 and P3 genomic regions. No significant segregation (<30% bootstrap support), however, was observed for the remaining isolates and this was perhaps due to the short sequence length of the 3' UTR(∼83nt). Based on these results, it appeared that sequences of genes at the 3' half of the EV-71 genome contributed to the multiple and diverse EV71 subgenotypes and these genes showed high similarity to different HEV-A viruses.

The incongruent phylogenies and simplot similarity analyses imply that recombination has played an evident role in the evolution of C4 EV71 viruses. C4a and C4b clearly contained sequences in the non-capsid region that are also present in CVA4,14,16, suggesting that these three HEV-A strains and C 4 subgenotype viruses of EV71 have a shared evolutionary history, despite their lack of similarity in the capsid region. However, the exact recombination counterpart of HEV-A could not be found because there is not sufficient data regarding the P2 and P3 sequences of the HEV-A in China or any other part of the world, but it may be assumed that genetic exchanges had occurred when the HEV71 strain co-circulated with other HEV-A during that time period in China. In this study, we have already performed the Nucleotide acid and deduced amino acid sequence identities analysis, and Phylogenetic analysis based on 5′UTR, P1, P2, P3, 3′UTR region of the genome respectively. Both identities and phylogenetic analysis indicated that both C4a and C4b viruses had much higher sequence identities with EV71 in P1 region; while in the P2 and P3 regions, both C4a and C4b viruses had much higher sequence identities with CVA-16,14,4. Although we combined the simplot analysis, the identities and phylogenetic analysis to confirm the recombination of C4 EV71 from the prototype strains of CAV16, 14 and 4, it is still a hypothesized conclusion.

Species human enterovirus A (HEV-A) which include 11 members of the coxsackievirus A (CV-A) group; CVA2–8, CVA10, CVA12, CVA14, CVA16 and human enterovirus 71 (EV-71) are associated with several human diseases [1], [2]. CVA4 cause herpangina [2] and EV71, CVA14, CVA16 are highly associated with HFMD, and EV71 and CVA7 are occasionally associated with neurological diseases [20], [33]. Seiya Yamayoshi, et al reported that EV71, CVA7, CVA14 and CVA16, utilized the same cellular receptor SCARB2, a critical receptor common to all EV71 strains, for infection. Provided these SCARB2-dependent viruses sometimes co-circulate during an epidemic of HFMD [34], [35], these viruses might have a high potential to undergo an intertypic recombination by co-infection of a SCARB2-expressing cell in vivo. In this study, we provided the evidence for the intertypic recombination of C4 subgentype between EV71 and CVA16, 14, 4. Based on the published co-infection and the cellular receptor study, we proposed that C4a and C4b viruses are intertypic recombination viruses between EV71 and other HEV-A strains derived from CVA16 or CVA14, not CVA4, since CVA4 infected cells via the different cellular receptor pathway and associated mainly with different clinical outcome, herpangina [33]. The utilization of same cellular receptor between EV71 and CVA7 strains provided the possibility for the recombination between these two enteroviruses, which might appear as an emerging infectious pathogen, however, and may have unexpectedly high virulence, since both of them associated with neurological diseases. Careful and continuous surveillance of these viruses for the potential recombination is important for public health.

In this study, we provided the evidence confirming that these recombination C4 viruses have been occurred in China since 1998 and persistently circulated in China more than 14 years, and evolved into 2 major evolution lineages, C4a and C4b viruses during the decade. More and more severe neurological diseases and fatal cases have been caused by the intertypic recombinant C4a viruses throughout mainland China since 2007. A total of 5,034,764 HFMD cases including 61,582 severe and 1,894 fatal cases were reported to NNDRS during 2008–2011 in mainland of China [11], [17], with increase of the severe and fatal cases year by year. The recombination C4a EV71 viruses have been associated with more than 80% of the severe cases and 92% of the fatalities since 2008 [17]. The reason for the epidemic of large-scale outbreaks of HFMD with increasing morbidity and mortality has been one of the most important issues in biomedical research in recent years. In the present study, except for the surveillance gap in mainland China during 1999–2000 and 2004–2006, we obtained the full scope of the EV71 epidemic based on the complete genome analysis. Our present and previous data indicated that C4b viruses had been circulating in mainland China for 5 years since 1998 (C4b was prevalent from 1998 to 2003 but has now disappeared from mainland China), which rarely caused severe disease and death in HFMD patients, and replaced by C4a viruses causing the nationwide outbreak with higher morbidity and mortality and caused many severe and fatal HFMD patients since 2007. The acquisition of a segment of genes from CV-A16 or CVA14 by EV71 C4b viruses could have rendered the virus more fit to adapt to a new environment especially the host immunity. And then, C4b viruses continued to adapt to its hosts via nucleotide mutations, while such mutations often lead to the changes in the pathogenicity, patterns of prevalence, and clinical manifestations of HEV71 infection, complicating clinical diagnosis, et al. Hence it could be the reason why C4b was rapidly replaced by C4a viruses which remained higher prevalence with increasing neurovirulence and transmissibility in mainland China since 2007. Additionally, it is possible that the C4a EV71 strains currently circulating in mainland China associated with higher morbidity and mortality obtained an unidentified neurovirulence determinant(s) in the viral genome and became more neurovirulent than those that circulated previously. Because it has been reported that an increase in neurovirulence levels can be caused by point mutations or by genetic recombination between avirulent poliovirus vaccine strains and nonpolio enteroviruses [33]. Other research groups have identified the genetic differences between EV71 isolates from patients with severe and mild clinical disease and try to test the virulence phenotype of these recombinant viruses in the mouse model of infection via a reverse genetic approach. And our research team is currently using reverse genetics methods to identify the key nucleotide and amino acid differences between evolutionary branches C4a and C4b in order to discover the important determinants of neurovirulence and the possible reasons for the repeated outbreaks of HFMD in China in recent years. Our comprehensive study on the complete genome of C4 subgenotype of EV71 is significant for the prevention and control, vaccine development of EV71 in the world. However, genetic studies alone are not significant enough for vaccine development. It is critical to study antigenic variations for selecting vaccine strains [36].

## Materials and Methods

### Viruses and Sequence

This study did not involve human participants or human experimentation; the only human materials used were stool samples, throat swab samples, and vesicles collected from HFMD patients at the instigation of the Ministry of Health P. R. of China for public health purposes, and written informed consent for the use of their clinical samples was obtained from all patients involved in this study. This study was approved by the second session of the Ethics Review Committee of the Chinese Center for Disease Control and Prevention. The EV71 strains used in this study were isolated between 2002–2003 and 2007–2009 from stool, throat swabs, or vesicles from HFMD patients from different geographical locations in the Shanghai, Chongqing, Anhui, Shandong and Henan provinces of China (Table S1). Viruses were isolated from original clinical specimens by propagation in RD (human rhabdomyosarcoma) cells by conventional methods and then sequenced. To investigate the full genetic characterization of the complete genome of EV71 in Mainland China, 186 EV71 complete genome available from GenBank were downloaded and aligned with 16 full-length of EV71 determined in the present study, and then, 65 sequences from GenBank (based on the genetic diversity, those sequences identical and <2% were cleared out) and 16 sequences from this study were chosen to do the further phylogenetic and recombination analysis (Table S1).

### Determination of the Full-length Genome Sequencing of EV71

Viral RNA was extracted from the viral isolates using a QIAamp Viral RNA Mini Kit (Qiagen, Valencia, CA, USA) and stored at -80°C until further use. The full-length genomes of 16 EV71 strains from the HFMD patients were amplified and sequenced. The viral RNA was converted to cDNA by a random priming strategy. The cDNA was amplified using the primers designed by multiple alignments of EV71 genomes available in GenBank database(Table S2). PCR products obtained were purified using the QIAquick Gel extraction kit (Qiagen). The amplicons were then bi-directionally sequenced using an ABI PRISM 3100 genetic analyzer (Applied Biosystems). 5′-segment sequences were determined by using a 5′-rapid amplification of cDNA ends (RACE) core set (Takara Biomedicals), according to the manufacturer’s instructions.

### Phylogenetic and Bioinformatics Analysis

Sequencing analysis overlapping DNA sequences with at least 85% sequence homology and a minimum of 20 overlaps were assembled into contigs to generate consensus sequences using Sequencher version 4.0.5 (Gene Codes Corporation, USA). The consensus sequences were aligned against other EV71 complete genome sequences retrieved from the GenBank (Table S1) using MEGA5.05 [37]. 65 completely sequenced EV71 isolates available in the GenBank and the 16 isolates obtained from the present study were used for construction of phylogenetic trees. The genetic distance was determined by a pairwise estimation of the sequences percent divergence. Positions with gaps were included, transition and transversion ratio was fixed at 10 and corrections for multiple substitutions were made. These options were chosen to take into account the ambiguous part of the alignments and to correct for more than one substitutions happening at many sites that may underestimate the actual genetic distances [38]. Phylogenetic trees presented here were constructed using MEGA 5.05 with neighbour-joining method [37]. The strength of the phylogenetic trees was estimated by bootstrap analyses using 1000 random samplings. A bootstrap value of ≥80% indicates a strong support for the tree topology [39]. Amino acid sequences were examined after stripping the 5' UTR and 3' UTR sequences and consensus sequences for each EV71 genotype were established.

### Recombination Analysis

Two nucleotide alignments were generated using the MEGA 5.05 [37]. The first alignment contains the genome sequences of a C4b evolutionary branch EV71 strain (SH-17/SH/CHN/2002), the oldest strains of each subgenotype of EV71 and HEV-A prototype strains (CVA-2, 3, 4,5, 6, 7, 8, 10, 12, 14, 16, EV71, 76, 89, 90, 91 and 92); The second contains the genome sequences of a C4a evolutionary branch EV71 strain (HeN09-17/HeN/CHN/2009), the oldest strains of each subgenotype of EV71 and HEV-A prototype strains. Once aligned, similarity plot and bootscan analysis were performed using Simplot program (version 3.5.1; Stuart Ray, Johns Hopkins University, Baltimore, Maryland, USA) [39]. To demonstrate the recombination analysis clearly, 13 viruses were selected based on identity analysis for Figure 3.

### Nucleotide Sequence Accession Numbers

The 16 sequences reported in this study were deposited in the GenBank sequence database, accession numbers: EU703812 to EU703814; JX678874-JX678886.

## Supporting Information

Table S1 List of 186 HEV71 strains.
(DOC)

Table S2 Primers for RT-PCR, Sequencing and RACE.
(DOCX)

Table S3 The difference between this study and other studies (ref. 19, 20, and 27) to identify different break point and parental strains of EV71 C4a and C4b recombinants.
(DOCX)

Figure S1 Phylogenetic trees showing the relationships amongst HEV-A isolates using the different genomic regions. The neighbour-joining trees were constructed from alignment of the 2A (a), 2B (b), 2C3A3B (c), and 3C3D (d) genomic region, respectively. The percentage of bootstrap (percentage of 1000 pseudoreplicate datasets) replicates supporting the trees are indicated at the nodes; for clarity, only values over 80% are shown. The branch lengths are proportional to the genetic distances corrected using Kimura-two-parameter substitution model.
(PPT)
