# Supplementary material for: Complete Genome Analysis of the C4 Subgenotype Strains of Enterovirus 71: Predominant Recombination C4 Viruses Persistently Circulating in China for 14 Years
Source: PLoS One. 2013 Feb 18;8(2):e56341. doi: 10.1371/journal.pone.0056341 (PMC3575343; doi:10.1371/journal.pone.0056341)
Supplement: Table S1 — List of 186 HEV71 strains. (DOC) [file pone.0056341.s001.doc]

TableS1. List of the HEV71 strains used to generate the HEV71 phylogenetic dendrograms.

| Strain name | Source | Genbank Accession Number | Genotype | The year of isolation | The place of isolation | Associated pathological conditions |
| --- | --- | --- | --- | --- | --- | --- |
| 09-17/HeN/CHN/2009 | GenBank | HM212466 | C4a | 2009 | Henan, China | Mild |
| M188-1181F/HeN/CHN/2009 | GenBank | JN256064 | C4a | 2009 | Henan, China | Severe |
| Henan/399/HeN/CHN/2010 | GenBank | HM245928 | C4a | 2010 | Henan, China | NA |
| 09-1/HeN/CHN/2009 | GenBank | HM212465 | C4a | 2009 | Henan, China | NA |
| BJ398/BJ/CHN/2009 | GenBank | HM002489 | C4a | 2009 | Beijing, China | NA |
| Shanghai27/SH/CHN/2009 | GenBank | HQ891923 | C4a | 2009 | Shanghai, China | NA |
| HN318/HeN/CHN/2011 | GenBank | JQ639384 | C4a | 2011 | Henan, China | NA |
| DTID/ZJU-62/ZJ/CHN/2008 | GenBank | FJ158600 | C4a | 2008 | Zhejiang, China | NA |
| EV71/JN200804/SD/CHN/2008 | GenBank | HQ825317 | C4a | 2008 | Shandong, China | NA |
| EV71/Jinan1006/SD/CHN/2010 | GenBank | JQ074190 | C4a | 2010 | Shandong, China | NA |
| FY08-C30-P2/FY/AH/CHN/2008 | GenBank | GU198367 | C4a | 2008 | Anhui, China | NA |
| FY23/AH/CHN/2008 | GenBank | EU812515 | C4a | 2008 | Anhui, China | NA |
| MP10/BJ/CHN/2010 | GenBank | HQ712020 | C4a | 2010 | Beijing, China | Mild |
| MP9-1/BJ/CHN/2011 | GenBank | JF894381 | C4a | 2011 | Beijing, China | NA |
| EV71/Ningbo065/ZJ/CHN/2010 | GenBank | JF830007 | C4a | 2010 | Zhejiang, China | NA |
| NB/2010/01/ZJ/CHN/2010 | GenBank | JN001860 | C4a | 2010 | Zhejiang, China | NA |
| Fuyang-0805/AH/CHN/2008 | GenBank | FJ439769 | C4a | 2008 | Anhui, China | Mild |
| 2010FJLY008/AH/CHN/2010 | GenBank | HQ426649 | C4a | 2010 | Anhui, China | NA |
| JN200803/SD/CHN/2008 | GenBank | JF913464 | C4a | 2008 | Shandong, China | NA |
| BJ09/07 BJ/CHN/2009 | GenBank | JQ319054 | C4a | 2009 | Beijing, China | NA |
| HFMD Severe/Jingdezhen/JX/CHN/2011 | GenBank | JQ806378 | C4a | 2011 | Jiangxi,China | Severe |
| LN009/LN/CHN/2010 | GenBank | HQ407557 | C4a | 2010 | Liangning,China | NA |
| NBChina01/ZJ/CHN/2010 | GenBank | HQ828086 | C4a | 2010 | Zhejiang, China | NA |
| G288-927F/HeN/CHN/2009 | GenBank | JN256059 | C4a | 2009 | Henan, China | Fatal |
| EV71/HENAN/DC/HeN/CHN/2010 | GenBank | HQ325852 | C4a | 2010 | Henan, China | NA |
| EV71/Lanzhou01/GS/CHN/2009 | GenBank | GU396280 | C4a | 2009 | Gansu,China | NA |
| SHZH/08/HFMD Severe/SHZH/CHN/2008 | GenBank | FJ607338 | C4a | 2008 | Shenzhen,China | NA |
| Luoyang/HeN/CHN/2011 | GenBank | JN020147 | C4a | 2011 | Henan, China | NA |
| Nanyang/HeN/CHN/2011 | GenBank | JN052925 | C4a | 2011 | Henan, China | NA |
| Kaifeng/HeN/CHN/2010 | GenBank | JQ517316 | C4a | 2010 | Henan, China | NA |
| EV71/Henan/106/HeN/CHN/2009 | GenBank | HQ998852 | C4a | 2009 | Henan, China | NA |
| EV71/Zhejiang08/ZJ/CHN/2008 | GenBank | EU864507 | C4a | 2008 | Zhejiang, China | NA |
| Henan10-08/HeN/CHN/2010 | GenBank | GU366191 | C4a | 2010 | Henan, China | NA |
| 518-03F/SD/CHN/07 | GenBank | EU753365 | C4a | 2007 | Shandong, China | Severe |
| Anhui1-09/AH/CHN/2009 | GenBank | GQ994988 | C4a | 2009 | Anhui, China | Severe |
| 036-2009/SH/CHN/2009 | GenBank | FJ713137 | C4a | 2009 | Shanghai, China | NA |
| TW/70516/08/TW/2008 | GenBank | GQ231933 | C4a | 2008 | Taiwan | Mild |
| H8-1/HeN/CHN/2008 | GenBank | JQ681218 | C4a | 2008 | Henan, China | NA |
| BJ08-Z004-3/BJ/CHN/2008 | GenBank | FJ606447 | C4a | 2008 | Beijing, China | NA |
| Henan2-09/HeN/CHN/2009 | GenBank | GQ994992 | C4a | 2009 | Henan, China | Severe |
| TW/1956/05/TW/2005 | GenBank | GQ231926 | C4a | 2005 | Taiwan | Severe |
| 540V/VNM/2005 | GenBank | JQ965759 | C4a | 2005 | Vietnam | NA |
| TW/2728/04 /TW/2004 | GenBank | GQ231929 | C4a | 2004 | Taiwan | Severe |
| EV141 06/CANADA/2006 | GenBank | HQ647171 | C4a | 2006 | Canada | NA |
| SHZH03/CHN/2003 | GenBank | AY465356 | C4b | 2003 | Shenzhen,China | NA |
| AFP2001064/EV71/GX/CHN/2001 | GenBank | JQ742001 | C4b | 2001 | Guangxi,China | NA |
| SHZH98/SHZH/CHN/1998-C4 | GenBank | AF302996 | C4b | 1998 | Shenzhen,China | Mild |
| N3340/TW/2002 | GenBank | EU131776 | C4b | 2002 | Taiwan | NA |
| EV71/GDFS/3/GD/CHN/2008 | GenBank | FJ194964 | C4b | 2008 | Guangdong,China | NA |
| Chongqing1-09/CQ/CHN/2009 | GenBank | GQ994989 | C4b | 2009 | Chongqing,China | Mild |
| 121/SHZH/CHN/2008 | GenBank | FJ607337 | C4a | 2008 | Shenzhen,China | Fatal |
| KM186/YN/CHN/2009 | GenBank | HQ423143 | C4a | 2009 | Yunnan,China | NA |
| 2007-07364/TW/2007 | GenBank | EU527983 | C5 | 2007 | Taiwan | NA |
| S40221/SAR/2000 | GenBank | DQ341358 | C1 | 2000 | Malaysia | NA |
| Tainan/5746/98/TW/1998 | GenBank | AF304457 | C2 | 1998 | Taiwan | Mild |
| 03/KOR/2000 | GenBank | DQ341356 | C3 | 2000 | South Korea | NA |
| BrCr/USA/1970 | GenBank | U22521 | A | 1970 | USA | Severe |
| EV71-Hubei-09/HuB/CHN/2009 | GenBank | GU434678 | B5 | 2009 | Hubei,China | NA |
| 3799-SIN/1998 | GenBank | DQ341354 | B3 | 1998 | Singapore | NA |
| 10857/NED/1966 | GenBank | AB575912 | B0 | 1966 | Netherlands | NA |
| Nagoya/JAN/1973 | GenBank | AB482183 | B1 | 1973 | Japan | NA |
| MS/7423/87/USA/1987 | GenBank | U22522 | B2 | 1987 | USA | Severe |
| SB2864/SAR/2000 | GenBank | DQ341366 | B4 | 2000 | Malaysia | NA |
| S19841/SAR/2003 | GenBank | DQ341363 | B5 | 2003 | Malaysia | NA |
| 2007-08747/TW/2007 | GenBank | EU527985 | B5 | 2007 | Taiwan | NA |
| EV71/Xiamen/FJ/CHN/2009 | GenBank | JN964686 | B5 | 2009 | Fujian,China | NA |
| G10/SOA/1951 | GenBank | U05876 | / | 1951 | South Africa | NA |
| SH-6/SH/CHN/2002 | This study | JX678886 | C4b | 2002 | Shanghai, China | Mild |
| SH-17/SH/CHN/2002 | This study | JX678885 | C4b | 2002 | Shanghai, China | Mild |
| CQ03-1/CQ/CHN/2003 | This study | JX678874 | C4b | 2003 | Chongqing,China | Mild |
| SD07-1/SD/CHN/2007 | This study | JX678882 | C4a | 2007 | Shandong, China | Severe |
| FY17.08-1/AH/CHN/2008 | This study | EU703812 | C4a | 2008 | Anhui, China | Fatal |
| FY17.08-2/AH/CHN/2008 | This study | EU703813 | C4a | 2008 | Anhui, China | Fatal |
| FY17.08-3/AH/CHN/2008 | This study | EU703814 | C4a | 2008 | Anhui, China | Fatal |
| FY17.08-4/AH/CHN/2008 | This study | JX678875 | C4a | 2008 | Anhui, China | Mild |
| FY17.08-5/AH/CHN/2008 | This study | JX678876 | C4a | 2008 | Anhui, China | Mild |
| FY17.08-6/AH/CHN/2008 | This study | JX678877 | C4a | 2008 | Anhui, China | Severe |
| FY17.08-7/AH/CHN/2008 | This study | JX678878 | C4a | 2008 | Anhui, China | Severe |
| FY17.08-8/AH/CHN/2008 | This study | JX678879 | C4a | 2008 | Anhui, China | Severe |
| SD09-14/SD/CHN/2009 | This study | JX678883 | C4a | 2009 | Shandong, China | NA |
| SD09-21/SD/CHN/2009 | This study | JX678884 | C4a | 2009 | Shandong, China | Severe |
| HeN09-1/HeN/CHN/2009 | This study | JX678880 | C4a | 2009 | Henan, China | Mild |
| HeN09-17/HeN/CHN/2009 | This study | JX678881 | C4a | 2009 | Henan, China | Mild |
