# Supplementary material for: Complete Genome Analysis of the C4 Subgenotype Strains of Enterovirus 71: Predominant Recombination C4 Viruses Persistently Circulating in China for 14 Years
Source: PLoS One. 2013 Feb 18;8(2):e56341. doi: 10.1371/journal.pone.0056341 (PMC3575343; doi:10.1371/journal.pone.0056341)
Supplement: Table S2 — Primers for RT-PCR, Sequencing and RACE. (DOCX) [file pone.0056341.s002.docx]

**Table S2: Primers for RT-PCR, Sequencing and RACE.**

| **Name** | **Primer sequence（5’-3’）** | **Nucleotide Position** | **Orientation** |
| --- | --- | --- | --- |
| EV71-1S | TTAAAACAGCCTGTGGGTTG | 1-20 | Forword |
| EV71-2A | ATCCAATAGCTATATGGTAA | 619-639 | Reverse |
| EV71-3S | AACTCTGCAGCGGAACCGAC | 533-552 | Forword |
| EV71-4A | AACCTATTCACCGAAACATC | 1128-1147 | Reverse |
| EV71-5S | ATAGTTGGTTATGGTGAGTG | 1053-1072 | Forword |
| EV71-6A | ATTGGAGCCAGAGTGAT | 1665-1681 | Reverse |
| EV71-7S | TGATTCTGCCTTGAACCAYT | 1568-1587 | Forword |
| EV71-8A | TGAGTGTTGCTGATCCATGG | 2229-2248 | Reverse |
| EV71-9S | TCATAGCCTACACACCACCA | 2116-2135 | Forword |
| EV71-10A | CCTGTTATGTCTATGTCCCA | 2772-2791 | Reverse |
| EV71-11S | GGATTAGTTGGAGAGATAGA | 2619-2635 | Forword |
| EV71-12A | TGACGTGCTTCATCCTC | 3206-3222 | Reverse |
| EV71-13S | GTCCAAACAACATGATG | 3121-3137 | Forword |
| EV71-14A | TCAGACACACCCTGCTCCAT | 3780-3799 | Reverse |
| EV71-15S | TCAGAACCAGGAGATTGYGG | 3650-3670 | Forword |
| EV71-16A | AAGACACATTGCCAAAC | 4313-4329 | Reverse |
| EV71-17S | CAGTAAATTCATCGATTGGC | 4160-4179 | Forword |
| EV71-18A | CACTGTTGGCACTATGATGT | 4762-4782 | Reverse |
| S4662 | ACCGTAGACTTCATTCCACCA | 4662-4683 | Forword |
| A5794 | TGGTGCGATGGGTAGGCTTA | 5774-5794 | Reverse |
| EV71-23S | ATCACCAAGTTCATACCAGA | 5653-5672 | Forword |
| EV71-24A | CTGTATGGATATCCTGCACT | 6289-6308 | Reverse |
| EV71-25S | GCGGCCCTCCATTATGC | 6175-6191 | Forword |
| EV71-26A | GGCATTCCACCAAGAACACA | 6792-6811 | Reverse |
| EV71-27S | CTCAGCCCAGTGTGGTTCAG | 6667-6686 | Forword |
| EV71-28A | GCTATTCTGGTTATAACAAA | 7403-7422 | Reverse |
| S7146 | ATGGACCAAGGACGCACGAA | 7146-7176 | Forword |
| EV71-OligoDT | TTTTTTTTTTGCTATTCTGGT | NA |  |
| EV71-194 | TCAGTCCGGGGAAACAGAAGTG | 173-194 | Reverse |
| EV71-276 | CATGGTGTTACTAGGTTTTCCGAAG | 252-276 | Reverse |
