# Supplementary material for: Complete Genome Analysis of the C4 Subgenotype Strains of Enterovirus 71: Predominant Recombination C4 Viruses Persistently Circulating in China for 14 Years
Source: PLoS One. 2013 Feb 18;8(2):e56341. doi: 10.1371/journal.pone.0056341 (PMC3575343; doi:10.1371/journal.pone.0056341)
Supplement: Table S3 — The difference between this study and other studies (ref. 19, 20, and 27) to identify different break point and parental strains of EV71 C4a and C4b recombinants. (DOCX) [file pone.0056341.s003.docx]

T**ableS3**: The difference between this study and other studies (ref. 19, 20, and 27) to identify different break point and parental strains of EV71 C4a and C4b recombinants.

|  | **This study** | | **Ref. 19 :Yoke-Fun C, et al. (2006)** | | | **Ref. 20 :Huang SC, et al. (2008)** | | **Ref. 27: Yip CC, et al. (2010)** | | |
| --- | --- | --- | --- | --- | --- | --- | --- | --- | --- | --- |
| Qeury sequence | C4a:HeN09-17/HeN/CHN/2009  C4b: SH-17/SH/CHN/2002 | | C4b: ShenZhen98 | | | C4b:N3340-TW-02 | | C4a:SZ/HK08-5 | | |
| Break point | C4a：1-3771；  C4b：1-3771 | C4a：3772-7328；  C4b：3772-7328 | 1-355,3634-4148,  6770-7199 | 378-3632 | 4157-6752 | P1 regionA:3716-3781 | 2B-3B:5752-5776 | 1-3600 | 3600-5430 | 5430-7408 |
| parental strains | EV71 subgenotype C | CVA16，CVA14，CVA4 | EV71 genotype A | EV71 subgenotype C2 | CVA16 | EV71 genotypeC | EV71 genotypeB | EV71 genotypeC | EV71 genotypeB | CA16/G10 |
