# Supplementary figures and images for: Complete Genome Analysis of the C4 Subgenotype Strains of Enterovirus 71: Predominant Recombination C4 Viruses Persistently Circulating in China for 14 Years
Source: PLoS One. 2013 Feb 18;8(2):e56341. doi: 10.1371/journal.pone.0056341 (PMC3575343; doi:10.1371/journal.pone.0056341)

## Slide 1
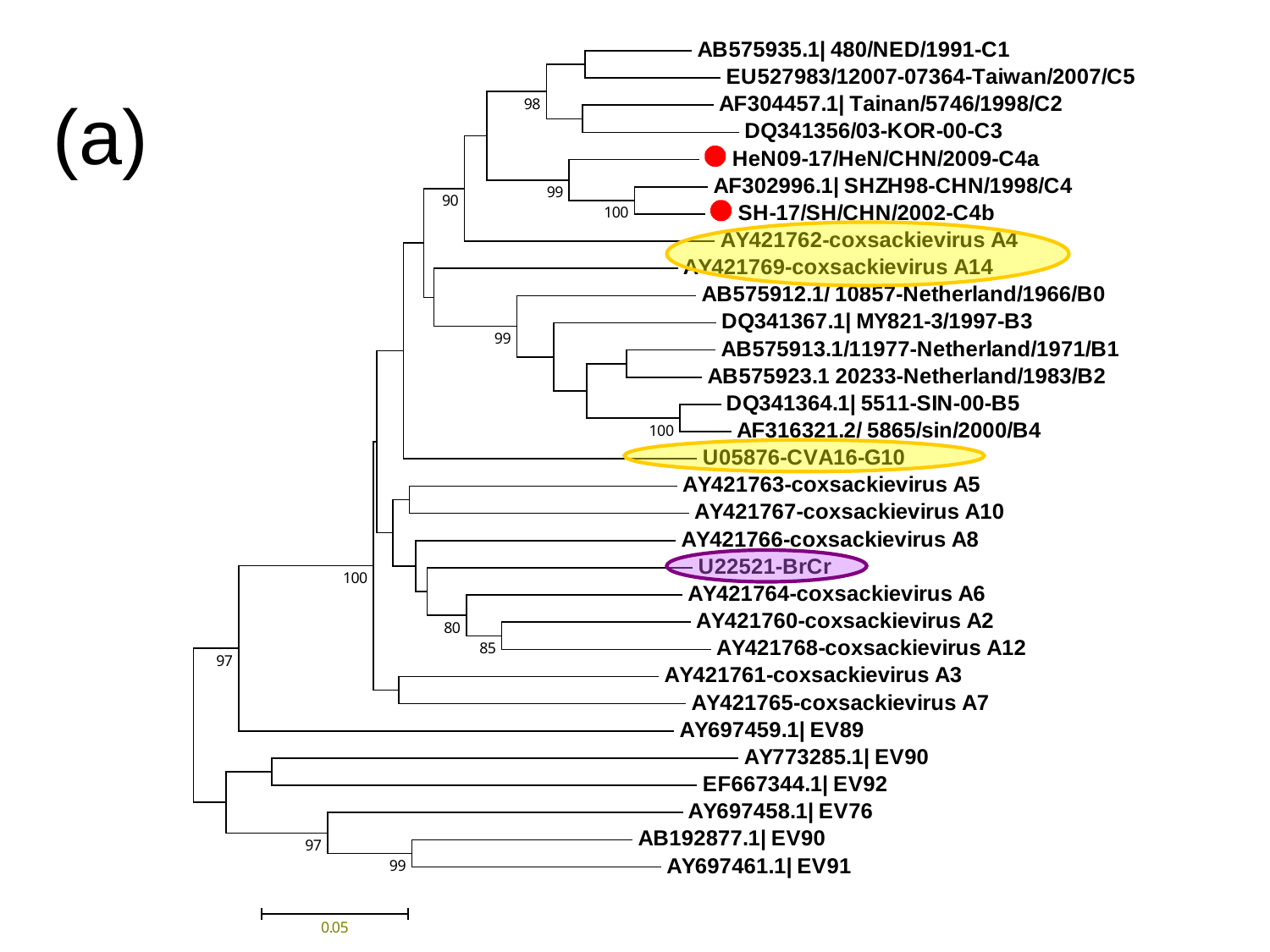

# (a)

## Slide 2
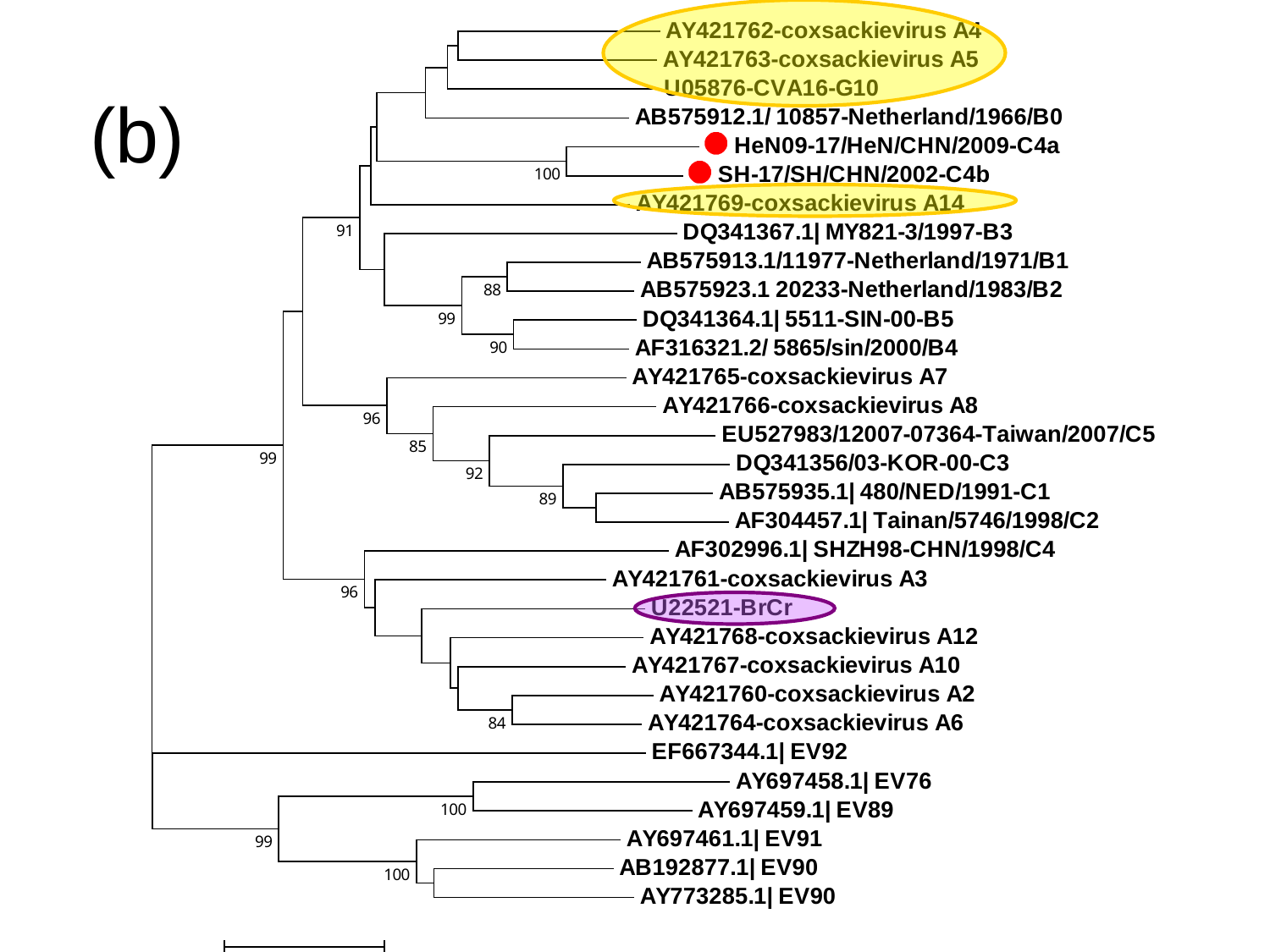

# (b)

## Slide 3
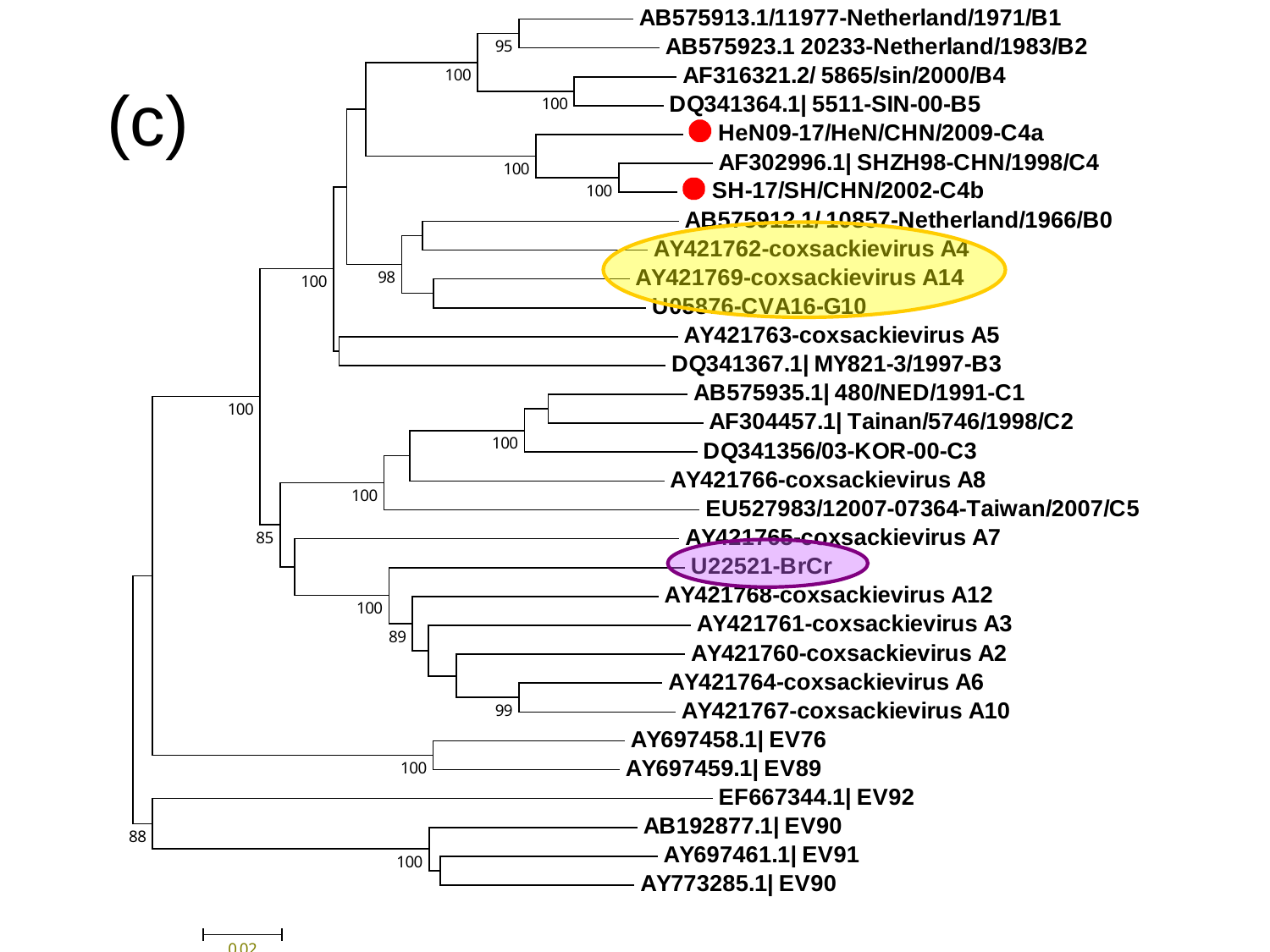

# (c)

## Slide 4
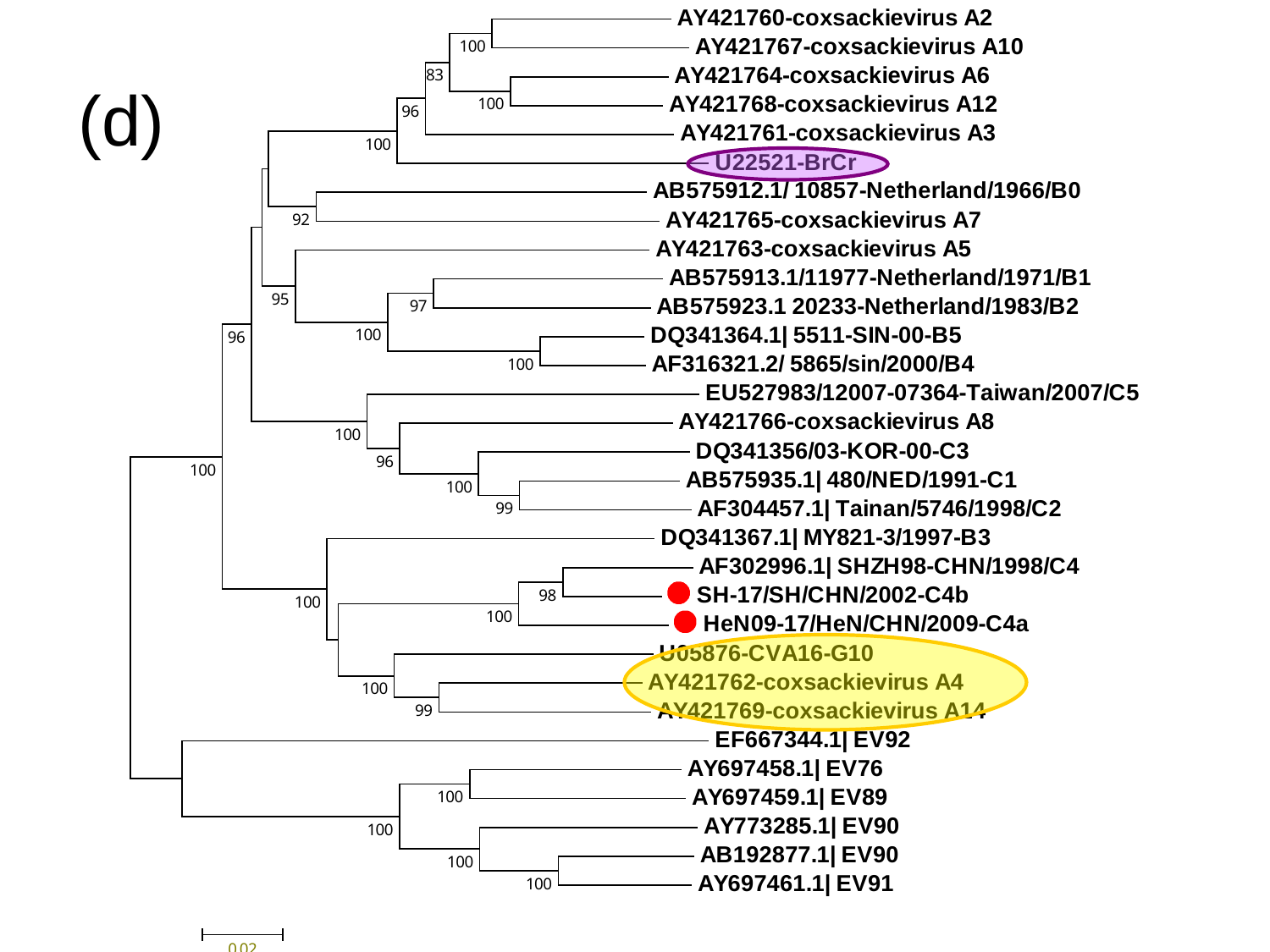

# (d)

Supplement: Figure S1 — Phylogenetic trees showing the relationships amongst HEV-A isolates using the different genomic regions. The neighbour-joining trees were constructed from alignment of the 2A (a), 2B (b), 2C3A3B (c), and 3C3D (d) genomic region, respectively. The percentage of bootstrap (percentage of 1000 pseudoreplicate datasets) replicates supporting the trees are indicated at the nodes; for clarity, only values over 80% are shown. The branch lengths are proportional to the genetic distances corrected using Kimura-two-parameter substitution model. (PPT) [file pone.0056341.s004.ppt]
